# Urethritis Caused by Novel *Neisseria meningitidis* Serogroup W in Man Who Has Sex with Men, Japan

**DOI:** 10.3201/eid2009.140349

**Published:** 2014-09

**Authors:** Kayoko Hayakawa, Ichiro Itoda, Ken Shimuta, Hideyuki Takahashi, Makoto Ohnishi

**Affiliations:** Shirakaba Clinic, Tokyo, Japan (K. Hayakawa, I. Itoda);; National Center for Global Health and Medicine, Tokyo (K. Hayakawa);; National Institute of Infectious Diseases, Tokyo (K. Shimuta, H. Takahashi, M. Ohnishi)

**Keywords:** Neisseria meningitidis, serogroup W, urethritis, men who have sex with men, MSM, sequence type 11, ST11, electrophoretic type 37, ET37, ST11/ET37 complex, bacteria, Japan

**To the Editor:** We report a case of urethritis caused by a novel multilocus sequence type (ST), 10651, of the ST11/electrophoretic type (ET)–37 complex *Neisseria meningitidis* serotype W. The patient was a man who has sex with men. We also report on the patient’s male partner, who was colonized with the same bacteria.

In March 2013, a 33-year-old Japanese man sought medical care at Shirakaba Clinic (Tokyo) after experiencing a urethral discharge for 4 days. The man was HIV positive (CD4 count 649 cells/μL) but was not receiving antiretroviral therapy. Physical examination showed a mucous urethral discharge. Gram staining of a sample revealed many gram-negative diplococci phagocytosed by polymorphonuclear leukocytes. Eleven days before seeking care, the patient had oral and anal intercourse with his male partner. A diagnosis of suspected urethritis caused by *Neisseria gonorrhoeae* was made, and a sample of the urethral discharge was sent for culture and testing (Strand Displacement Amplification) for *N. gonorrhoeae* and *Chlamydia trachomatis*. The patient was intravenously administered a single dose of ceftriaxone (1 g) (intramuscular administration of ceftriaxone is not approved in Japan). He was also given a single dose of azithromycin (1g orally) for possible *C. trachomatis* urethritis (*1*). 

Six days after receiving treatment, the patient showed improvement. Results of the Strand Displacement Amplification test were negative for *N. gonorrhoeae* and *C. trachomatis*. Eight days after the patient received treatment, the culture for the urethral discharge sample was shown to be positive for *N. meningitidis*. Urine culture was negative 20 days after treatment.

The 33-year-old male partner of the case-patient was originally from the United States and had been living in Japan for 4 years. Because of his history of sexual contact with the case-patient, he was advised to undergo a screening test for HIV and *N. meningitidis*. The man underwent a physical examination at our clinic 40 days after the case-patient received treatment; findings were unremarkable, and the result for HIV testing done 2 days earlier was negative. Throat and urine samples were obtained for culture, and the man was intravenously administered ceftriaxone (1 g). The urine sample culture was negative, but the throat sample culture was positive for *N. meningitidis*. The throat culture result was negative 10 days after the patient’s treatment.

We performed cultures and tests to identify *N. meningitidis*, and we conducted multilocus sequence typing (MLST), serotyping, PorA typing, and pulsed-field gel electrophoresis (PFGE) as described elsewhere (*2*). Isolates from both men were identified as serotype W and PorA type P1.5, 2. MLST showed that the strains were ST10651 (genes analyzed: *aroE*:3, *adk*:4, *fumC*:3, *gdh*:8, *pdhC*:4, *pgm*:6, and *abcZ*:662). Although *abcZ*:662 was a novel allele, ST10651 belongs to the ST11/ET37 complex (*3*). We used PFGE with restriction enzyme *Nhe*I to compare the *N. meningitidis* strains from the case-patient and his partner; the isolates had the same PFGE pattern (Figure). Both isolates were confirmed to be a novel multilocus ST, 10651, of the ST11/ET37 complex; however, novel MLST types frequently occur. By using the E-test (Sysmex bioMérieux, Tokyo, Japan), we determined that the 2 isolates required the same minimum inhibitory concentrations (MICs) for the following antimicrobial drugs: penicillin (MIC 0.125 mg/L), ceftriaxone (MIC 0.004 mg/L), ciprofloxacin (MIC 0.004 mg/L), and azithromycin (MIC 0.25 mg/L) (*4*).

**Figure:**
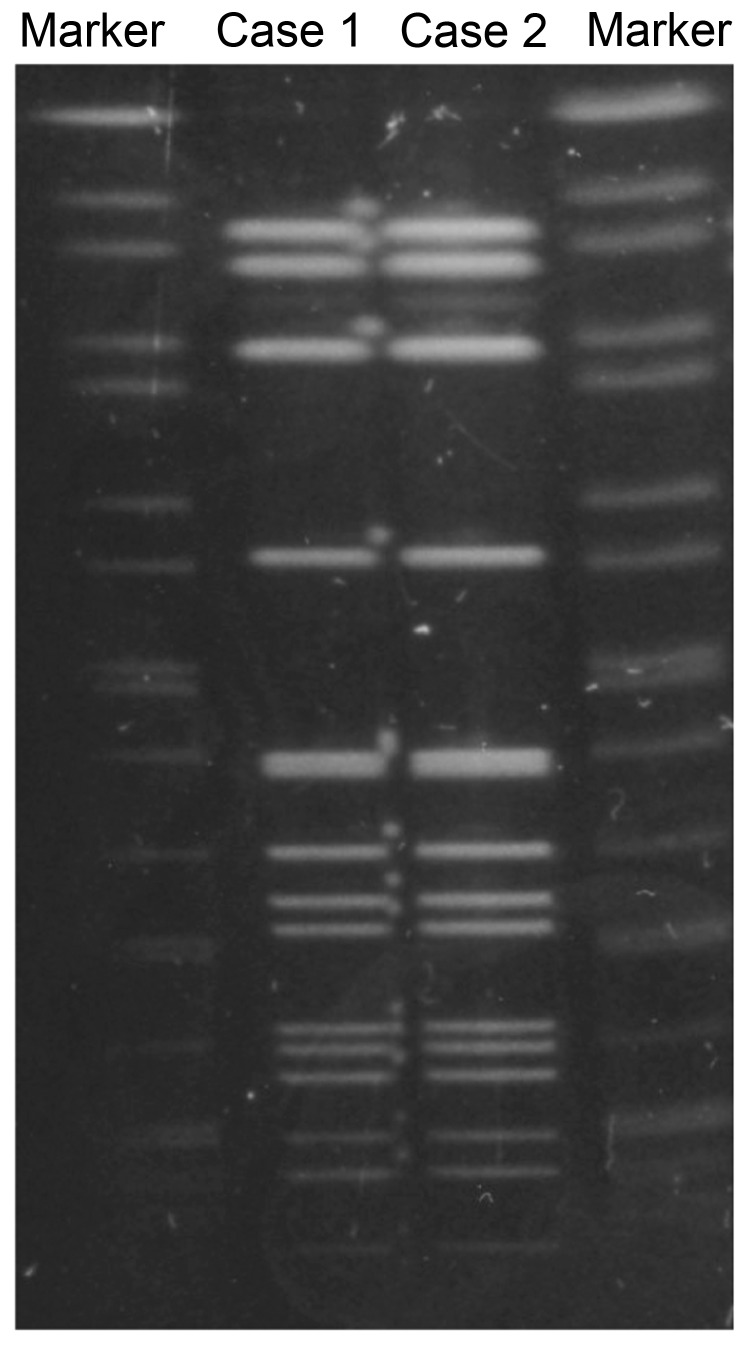
Pulsed-field gel electrophoresis (PFGE) patterns for *N. meningitidis* strains isolated from a man with urethritis (case 1) and his male sex partner (case 2), Japan. PFGE was performed with the restriction enzyme *Nhe*I. Results showed the same PFGE pattern for both isolates. *Salmonella* *enterica* serovar Braenderup strain H9812 was used as the PFGE size marker strain; it was digested with *Xba*l and resolved by PFGE.

Urethritis caused by *N. meningitidis* infection in men who have sex with men (MSM) has been reported, as has an association between urethritis and oral sex (*5*,*6*). Most previously reported urogenital isolates of *N. meningitidis* have belonged to serogroups B (*5*,*6*), Y (*5*,*6*), and C (*5*). Among 115 cases of *N. meningitidis* infection in Japan during the last 9 years, 22 (19.1%) were caused by serogroup B and 18 (15.7%) were caused by serogroup Y; only 3 (2.6%) cases were caused by serotype W (*7*).

*N. meningitidis* ST11/ET37 complex is a hyperinvasive lineage. During the 1990s, the serogroup C ST11/ET37 complex was prominent in Europe and North America. However, in 2000, an outbreak of *N. meningitidis* serotype W infections occurred among Hajj pilgrims (*8*), and this serotype has now spread worldwide (*3*,*8*).

Chemoprophylaxis is indicated for persons who have close contact with someone with invasive meningococcal infection (*9*), but there is uncertainty regarding the treatment of asymptomatic persons who have contact with someone with *N. meningitidis* urethritis. To avoid a reinfection cycle between the men in this study, we treated the asymptomatic, *N. meningitidis*–colonized male partner.

Since the early 2000s, and especially since 2012, outbreaks of invasive serogroup C, ST11/ET37 complex meningococcal disease causing high rates of death have been reported among MSM in the United States and Europe (*10*). These outbreaks have raised policy questions concerning vaccination recommendations for HIV-infected persons and for the MSM population (*10*). In Japan, meningococcal vaccination has not been officially approved, and neither of the men in this study had been vaccinated against *N. meningitidis*.

A diagnosis of urethritis is often based on Gram staining or nucleic acid amplification tests (*1*). However, Gram staining cannot differentiate *N. meningitidis* from *N. gonorrhoeae*, and amplification tests only detect *N. gonorrhoeae*. This practice makes it difficult to diagnose and access the number of cases of *N. meningitidis* urethritis.
